# Writing style of young physicians in the computer and internet era

**DOI:** 10.5116/ijme.534a.a3e2

**Published:** 2014-04-28

**Authors:** Gila Shilo, Lotan Shilo

**Affiliations:** 1 Culture and Humanities Division, Faculty of Society and Culture, Beit-Berl Academic College, Israel; 2 Sackler School of Medicine, Tel Aviv University, Israel

**Keywords:** Writing Style, residents, medical documents, curriculum, admission notes, discharge letter

## Abstract

**Objectives:**

The objective of the current study was to analyze written language of native Hebrew-speaking medical residents, as reflected in admission notes and discharge letters for patients admitted to medical wards in a 700-bed university hospital.

**Methods:**

Twenty admission notes and 20 discharge letters written by 40 native Hebrew- speaking residents with at least one year experience were analyzed. The residents worked in the Internal medicine departments of a 700-bed university hospital. Admission notes and discharge letters were randomly chosen for the analysis which was carried out using predefined linguistic criteria and the extent to which English or Latin terms were incorporated into Hebrew medical language such as the structure of sentences and paragraphs. (Complete list of the linguistic criteria can be found in the methods and results sections).

**Results:**

The most important findings were that the level of language was unexpectedly low. Many English or Latin medical terms were written using Hebrew letters. The creation of *‘new’* abbreviations was common. Sentences were telegraphic and lacked coherence, for example there were sentences written in internet language and short message service (SMS) messages. Texts were not organized and sometimes important details were missing.

**Conclusions:**

The writing style of medical residents should be improved substantially in order for them to be able to write coherently. One possible solution is to incorporate a course in writing into the medical school curriculum.

## Introduction

After graduating from medical school, residents have to compose several types of documents, such as admission notes, discharge letters, and letters to colleagues, consultants, primary care physicians, and patients. Additionally, they should be able to compose academic papers in order to enhance their professional satisfaction and academic achievements. When we searched the literature there was almost no mention of physicians’ writing style except in relation to academic writing,^1^ and relatively few publications addressed their correspondence with primary care physicians, consultants, and patients.^2^^-^^5^ Additionally, unlike verbal communication skills, writing skills do not receive much attention during residency.^6^In this study we sought to investigate the writing style of residents that use Internet, computers and smartphones extensively.The writing style of young physicians has changed tremendously over the last decades especially with the introduction of electronic records.^7^ The way the message and the content are conveyed today tends to be laconic, made up of short sentences, and containing many abbreviations. Experienced senior physicians argue that these changes are a result of the new generation being accustomed to working with computers and smartphones (sending text messages or replying to electronic mail). This parsimonious writing style was found to be especially common among the youngest people surveyed, ages 18-24.^8^ Experienced senior physicians, who write using higher language, claim that they are not able to substantially change the writing style of the younger generation of physicians. Instead, they tend to ignore the use of low level language and the communication style of the residents (personal communication).

## Methods

Forty discharge letters and admission notes written by different residents that who had finished at least one year of their residency were examined. Hebrew was the mother tongue of all. Text analysis criteria were chosen from textbooks and publications that describe the taxonomies commonly used.^9^^-^^12^ Accordingly, analysis of the texts was performed using linguistic, stylistic and rhetorical criteria that included reference to vocabulary, syntax, and text coherence. Mistakes that were found were classified into groups (vocabulary, syntax and rhetoric), and then further divided into subcategories. A sample of 10 admission notes and 10 discharge letters was arbitrarily chosen to calculate the occurrence of each type of mistake. The categories, subcategories and occurrences of each type of mistake are presented in Table 1. Further explanations and examples are presented and discussed. Patient's names and demographic details were removed from all documents before they were passed on to the researchers and the study was approved by the hospital ethics committee.

**Table 1:** Type and frequency of grammatical mistakes

Type of mistake		Occurrence (%)
Vocabulary mistakes		
	Use of Hebrew letters for writing English and Latin medical terms	52 (19)
	Disruption of exiting terms	10 (3)
	Inaccurate use of terms and words	26 (9)
	Use of uncommon forms	20 (6)
	Creation of "new" abbreviations in the medical language	20 (6)
	Spelling mistakes	6 (2)
Syntax mistakes		
	Lack of agreement with respect to singular and plural	16 (5)
	Incorrect use of propositions	26 (9)
	Punctuation in wrong places	18 (7)
	Lack of connectives	16 (5)
	Use of short, punctuated sentences	30 (11)
Textual mistakes		
	Problem with the chronological and logical organization of the text	8 (3)
	Introduction of irrelevant details	6 (2)
	Scattered and disorganized details	4 (1)
	Contradictions	6 (2)
	Repetition	4 (1)
	Lack of consistency	8 (3)
	Lack of details	18 (6)

## Results

The frequency of mistakes found in the admission notes and discharge letters are presented in Table 1. The three major mistake categories (Vocabulary, Syntax, Textual) are presented and below each one the mistake's subcategory. Beside the name of the subcategory the number of occurrences and the percentage from the total number of mistakes (in parenthesis) are presented.

### Vocabulary

In order for a message to be clear it is crucial to use clear and exact terms.

#### Writing English medical terms using Hebrew letters

The use of Hebrew letters and form for writing English terms is one of the most residents’ tendency to use English and Latin terms as if they are Hebrew terms. In these examples the words are written and pronounced as a noun in Hebrew. Examples include: *‘saturation’* becomes ‘*saturatia’*, *‘septic’* becomes *‘septi’*, and *‘catheterization’* becomes *‘cateterizatia’*, all written in the Hebrew alphabet. The main reason for these mistakes is the words are written and pronounced as a noun in Hebrew, and are actually incorporated to the Hebrew language.

#### Disruption of exiting terms

*‘Tachipnit’* instead of *‘thacipneic’* in English. Even if written as a noun in Hebrew it should have been written as *‘tachipnea’*. Another example is *‘liquefaction’* written in Hebrew *‘liquificacia’*, while such a term does not exist in the language. What should have been written was *‘containing fluid’*.

#### Inaccurate use of terms and words

For example *‘point was not measured’* instead of *‘point of maximal impact was not palpated’,* or *‘Low blood pressure was observed’* instead of *‘measured’*.

#### Using uncommon forms

Using the passive instead of the active form of words, a mistake in Hebrew language in the following examples: *‘seated on the floor’*, *‘not painful’*. Forms like this are uncommonly used in Hebrew language.

#### Creation of ‘new’ abbreviations that are not usually used in the medical language

There are abbreviations that are used daily such as BP for blood pressure and ECG for electrocardiogram but we found abbreviations such as ‘MNG’ as an abbreviation for multinodular goiter and ‘VSD’ for vascular surgery department (VSD is commonly used as abbreviation for ventricular septal defect). Improvised abbreviations such as these were found both for Hebrew and English terms.

#### Spelling mistakes

Both in Hebrew and in English words, such as *‘lecocitosis, dyverticolosis’* were written erroneously.

### Syntax

Lack of agreement with respect to singular and plural: *‘…normal breathing sound’* should be written *‘sounds’*, (plural is the correct form in Hebrew and English) or *‘bilateral rale’* instead of *‘bilateral rales’*.

#### Incorrect use of prepositions

*‘…helped with permanent urinary catheter’* actually have two mistakes in Hebrew: *‘helped’* (mistake in vocabulary) and *‘with’* (mistake in preposition).

#### Punctuation in wrong places

For example *‘was admitted because of constipation, vomiting’* instead of constipation and vomiting *‘on examination comfortable, not septic. Not tachypneic’.*

#### Lack of connectives

*‘The pain radiated to the back. Aroused from sleep. Deny shortness of breath’*. The sentence is fragmented; it might have been written: *‘the pain radiated to the back and aroused the patient from sleep. He denied shortness of breath, nausea or sweating’*.

#### Use of short, punctuated sentences

‘During hospitalization without fever’. ‘Breathing comfortably’. ‘Hemodynamically stable’. ‘Without leucocytosis’.

### Textual mistakes

Disruption of chronological and logical organization of the text: Writing in an unorganized way makes understanding the text difficult. For example: ‘S/P coronary angiography, LT main stenosis-25%, mid LAD stenosis 50-60%. The cardiac catheterization was performed during hospitalization because of chest pain. During the hospitalization ECG changes were observed and a decision to perform cardiac catheterization was made’. The correct order should be the reasons for performing the cardiac catheterization and only after that, the finding(s) during the procedure.

### Addition of irrelevant details

Relevance is one of the most important elements of coherence in a written text.^12^ Addition of irrelevant details makes it difficult for the reader to understand what the main ideas are. For example in the following paragraph, the physician wrote: *‘Hypertension for many years, treated by drugs. Moderate concentric hypertrophy was demonstrated by echocardiography that was performed in 6/2012. Since the beginning of biologic drug treatment the treatment with statins was stopped’,* (Irrelevant detail).

#### Scattered and unorganized details

The fact that details are not organized causes the writer to repeat information and facts that have been mentioned previously in the text. For example, this appeared in the discussion part of a discharge letter: *‘83 year old man suffering from severe dementia, decubitus ulcers stage 4 and is bedridden. The patient suffers from recurrent episodes of hematuria, and was admitted because of hematuria and fever. On physical examination fever of 38°C, leucocytes and positive nitrate in the urine that is coming out from the cystostomy tube. Another reason for fever may be the decubitus ulcers stage 4 the patient has in the regions of both trochanters and the sacrum’*.

The correct order should be first the presentation of demographic details and background, followed by the cause of admission, physical examination results, laboratory tests and discussion. In the example given the details are not organized as they should be and the information is scattered throughout, making the letter difficult to follow.

#### Contradictions

A paradox arises when there is a conflict between the facts and the details. For example it was written: *‘there was no fever during hospitalization, and no findings upon physical examination’*. However in the emergency room, a fever of 38.5°C was measured that was treated with Tylenol to lower the temperature and a systolic murmur was heard.

#### Repetition

Repetition can contribute to the coherence of the text when it helps to organize it. Another role of a repetition is to emphasize certain parts. However, it is unnecessary in the way it is used in the following example and also in the example above that deals with unorganized details. *‘Drug allergy-unknown. Allergies –unknown’*. This may indicate a lack of concentration on the part of the resident physician while writing or copying lines from a previous letter.^1^^,^^7^

### Lack of consistency

Lack of consistency was found in sentences and vocabulary. The following example is taken from *‘The instructions and recommendations’* in a discharge letter: *‘Follow up by her family physician’. ‘Regular medication’* instead of *‘Continuation of her regular medications’. ‘Consider an echocardiogram and/or Holter monitor in order to evaluate her arrhythmia’*. Should be *‘performance of echocardiogram and Holter monitor in order to check for arrhythmia’*.Another mistake here is that the arrhythmia was sinus arrhythmia that does not require further evaluation. This also serves as an example of lack of knowledge.

#### Lack of details

The causes can be lack of information and lack of knowledge. Writing a coherent text requires a certain amount of knowledge and familiarity with the details. Some of the texts we examined lacked details or revealed a lack of knowledge or both. Lack of details can be caused by lack of knowledge or by lack of attention (which is hardly surprising after a resident has worked for 23 hours straight). For example: *‘was admitted because of heartburn’*. It was not stated when exactly, how long the feeling has persisted, whether it was accompanied by sweating or diaphoresis. These details are important if the heartburn was a symptom, for example of myocardial infarction.Another example: *‘Lungs: vesicular breathing, rales over both lungs’.* It is not stated on which parts of the lungs. Another letter lacked details regarding professional and marital status. Examples for of lack of knowledge: *‘For one patient who suffered from pneumonia, basic blood tests and chest X-ray were not performed’*. *‘Bilateral cataract extraction dated…’* (Cataract extraction is always performed separately on each eye in order to verify the success of the operation before operating on the second eye).

## Discussion

From the results of the study we can conclude that the writing skill of medical residents needs improvement. Some of the mistakes that were found are not unique to medical writing such as lack of agreement with respect to singular and plural, spelling mistakes and punctuation in the wrong places. Several types of mistakes are probably typical of medical residents who are not native English speakers. The examples are in Hebrew but one would assume that a similar situation exists with other residents whose mother language is not English. For example, use of Hebrew letters for writing English or Latin medical terms.Many words found in the texts we examined are actually English or Latin terms that are written in Hebrew letters. It should be noted that diagnoses, names of drugs and laboratory test results are often written in Latin characters. Also, there are no rules when to use an English term in Hebrew letters and when to use Hebrew terms. For example relating to anatomy of the digestive tract: *‘mouth’, ‘esophagi*, and *‘small intestine’* will be written in Hebrew but *‘colon’* will be written as an English term but in Hebrew letters. Also the term *‘rectum’* will be used but written in Hebrew letters. In some cases an English term is transformed structurally to accord with Hebrew word formation and written in Hebrew. In other cases, the transformation is erroneous such as in the use of *‘tachipnit’* instead of *‘tachypneic’*. It is common to write names of medications in Hebrew letters, for example, ‘Zinnat’, the commercial name of an antibiotic used by one of the drug companies for cefuroxime acetyl is usually written in Hebrew letters in the text and English letters in the list of medications taken by the patient or recommended in the discharge letter. When writing prescriptions, the name of the drug may be generic or commercial, but it must always be written in English since this is required by law, and the pharmacist will not fill a prescription if the name of the drug is written in Hebrew. Regarding disease names, in some cases the Hebrew term is used, for example: *‘pneumonia’* will be called *‘daleket re’ot’*, but in the diagnosis list it will be written as pneumonia. It should be noted that the diagnosis list is always written in English or Latin terms since it is also used for coding purpose. Sometimes the same word is used in both languages, such as *‘cataract’*, but it is written using Hebrew letters. This is the most common mistake found in the corpus.The Academy of the Hebrew Language published a booklet of Hebrew words for medical terms, but most of the words are not used in clinical medicine, and the academy stopped distributing the booklet several years ago.Words are frequently used inaccurately, such as: *‘hudgam’* instead of *‘nir’ah’*, (*‘was demonstrated’* instead of *‘was seen’*), *‘yadu’a-na’asa’* (*‘is known’* instead of *‘was done’*). Perhaps this is part of the development of the new jargon in medical writing in Israel.Ambiguousness is one of the mistakes in vocabulary, as in this example: *‘Fever was not measured in the emergency department’*. In Hebrew this could mean that body temperature was not measured or that it was measured but found to be normal. In this particular case, it meant that the temperature was normal.Another problem that should be addressed is the use of abbreviations. Some of the abbreviations are in common use globally, such as ECG (electrocardiogram) and CABG (coronary artery bypass grafting). Residents also invented abbreviations, for example VSD (an accepted abbreviation for ventricular septal defect as mentioned before) was used as an abbreviation for vascular surgery department and MNG for multi nodular goiter. Abbreviations like that should not be used since most physicians will not understand their meaning.^13^Syntactic mistakes were frequent. Medical residents used short punctuated sentences unrelated to each other. This may cause lack of agreement with respect to singular and plural and incorrect use of prepositions. The causes may use of the *copy and paste* function during writing^14^ or the wish to write briefly and effectively. It may also be noted that there is a resemblance between these short sentences and phone text messages.Textual mistakes are also common. Despite the use of short sentences and the structured format of admission and discharge letters, poorly organized and incoherent text was found, especially in describing and discussing the current illness. Perhaps the reason lies in the fact that for these parts of the document there is no *drop down menu* as there is for the systems’ review or physical examination parts. The addition of irrelevant details, contradictions, repetition, lack of consistency and illogically organized text, all causes difficulty in understanding the written document. It is worth mentioning that writing guides have been published for engineers and also for physicians.^15^^,^^16^ Even the guide author mentioned the fact that *“the ability to write and speak in a concise, well-organized way is a skill not taught in medical schools”*.^16^One of the important causes of the mistakes found in the texts written by medical residents may be the extensive use of mobile devices. Medical students and residents rely heavily on their mobile devices for looking for guidelines, drug information, using of various medical applications, contact with peers and learning.^17^

## Conclusion

Our study demonstrates that the writing skills of resident physicians need improvement. We recommend that a course in medical writing be incorporated into the medical school curriculum. This course should include the organization of a text and the accurate use of medical terms and abbreviations. In addition, the correct use of punctuation and commas should be taught. Perhaps the most important part of this curriculum should be improving the student’s ability to write a summary and use *copy-paste* options (that is here to stay) in a way that will not produce incoherent texts.

## 

### Conflict of Interest

The authors declare that they have no conflict of interest.
